# Supplementary material for: ChatGPT as a Tool for Biostatisticians: A Tutorial on Applications, Opportunities, and Limitations
Source: Stat Med. 2025 Oct 23;44(23-24):e70263. doi: 10.1002/sim.70263 (PMC12548020; doi:10.1002/sim.70263)
Supplement: Supplementary file 1 — Data S1. Supporting Information. [file SIM-44-0-s001.zip › Supplementary Materials Additional.zip!/3.2_Diagnostic_Test/3.2_Diagnostic_Chat10.pdf]

ChatGPT - Diagnostic Testing

I would like to determine sensitivity and specificity of two diagnostic tests. I do have the diagnostic test outcomes, but I do not know the gold standard. What can I do?

Please, tell me more about the latent class analysis.

I have two populations with two different disease prevalences. I have two diagnostic tests which are conditional independent. I would like to get the log-likelihood of this setting.

What is the Hui-Walter paradigm?

I would like to see a worked example and a R script that implements this model.

I would like to add confidence intervals to the estimated sensitivities, specificities, and prevalences.

I would like to run a bootstrap version for robust confidence intervals.

I would like to reparametrize my model using a logistic parametrization for the prevalences, sensitivities, and specificities.

Please update the bootstrap function too.

Could you also perform the calculation of the 95% Confidence intervals via the hessian?

Please provide a comprehensive R code for the issues discussed in this session.

You've asked for a **comprehensive introduction and implementation** of **latent class analysis (LCA)** for estimating **sensitivity**, **specificity**, and **prevalence** without a gold standard, in the context of **two tests** and **two populations** (with different prevalences). This is known as the **Hui-Walter paradigm**.

I'll guide you step-by-step and provide a **complete R implementation** at the end.

📚 1️⃣ Conceptual Background

Problem:

- You have **two populations** (Pop 1 and Pop 2) with **different prevalences**.
- You have **two diagnostic tests**.
- You do **not have a gold standard**.

Goal:

- Estimate **sensitivities** and **specificities** of both tests.

- Estimate **prevalence** in both populations.
- Obtain **confidence intervals**.
- Implement **bootstrap** for robust inference.
- Implement **Hessian-based CIs**.
- Implement **logistic reparametrization**.

📚 2️⃣ Latent Class Analysis (LCA)

Idea:

You introduce a **latent variable** `D` (Disease status: 1 = diseased, 0 = not diseased).
Even though you do not observe `D`, you model the **observed joint test outcomes** as mixtures of "latent classes" (diseased and non-diseased).

📚 3️⃣ Hui-Walter Paradigm

Published in:

> Hui & Walter (1980). "Estimating the Error Rates of Diagnostic Tests".

Assumptions:

✅ Two populations with different prevalences
✅ Two diagnostic tests
✅ Conditional independence of tests **given the disease status**
✅ No gold standard available

📚 4️⃣ Likelihood Construction

Let:

- **Populations**: `k = 1, 2`
- **Disease prevalences**: π_1, π_2
- **Sensitivities**: Se_1, Se_2
- **Specificities**: Sp_1, Sp_2

Observed test outcomes:
Each subject can be in one of **4 possible test result combinations**:

Test 1	Test 2	Combination
+	+	1
+	-	2
-	+	3

Test 1	Test 2	Combination
-	-	4

For each population, you observe the **count of subjects** in each of these 4 categories.

Probabilities:

For population k:

- **Diseased (D=1)**: π_k
  - Pr(++ | D=1) = Se_1 * Se_2
  - Pr(+- | D=1) = Se_1 * (1 - Se_2)
  - Pr(-+ | D=1) = (1 - Se_1) * Se_2
  - Pr(-- | D=1) = (1 - Se_1) * (1 - Se_2)
- **Non-diseased (D=0)**: (1 - π_k)
  - Pr(++ | D=0) = (1 - Sp_1) * (1 - Sp_2)
  - Pr(+- | D=0) = (1 - Sp_1) * Sp_2
  - Pr(-+ | D=0) = Sp_1 * (1 - Sp_2)
  - Pr(-- | D=0) = Sp_1 * Sp_2
- **Total probabilities** in population k:
  `Pr( outcome ) = πₖ * Pr(outcome | D=1) + (1 - πₖ) * Pr(outcome | D=0)`

Log-likelihood

For each population and each outcome combination, we observe counts $n_{k,1}$, $n_{k,2}$, $n_{k,3}$, $n_{k,4}$.

We model:

```bash
LogLik = sum over populations k of:
  sum over categories j of:
    nₖ,ⱼ * log(Prⱼₖ)
```

📚 5️⃣ R Implementation

Now I will provide a **complete, modular R script** that includes:

✅ **Model definition**
✅ **Maximum likelihood estimation**
✅ **Bootstrap for robust CI**
✅ **Logistic reparametrization**
✅ **Hessian-based CI**

🔥 Full R Code: Hui-Walter Latent Class Model

```r
# Load necessary libraries
library(stats4)
library(numDeriv)

# --- Data Input: Counts per test outcome per population ---

# Example synthetic data:
# Format: c(n_++, n_+-, n_-+, n_--)
# You should replace these with YOUR observed counts

# Population 1 (high prevalence)
counts1 <- c(50, 10, 15, 25)

# Population 2 (low prevalence)
counts2 <- c(10, 5, 20, 65)

# --- Model: Negative Log-Likelihood ---

negloglik <- function(
  logit_pi1, logit_pi2,
  logit_se1, logit_sp1,
  logit_se2, logit_sp2
) {
  # Transform parameters to (0,1) via logistic
  pi1 <- plogis(logit_pi1)
  pi2 <- plogis(logit_pi2)
  se1 <- plogis(logit_se1)
  sp1 <- plogis(logit_sp1)
  se2 <- plogis(logit_se2)
  sp2 <- plogis(logit_sp2)

  # Outcome probabilities (4 categories)
  prob_cat <- function(pi) {
    c(
      pi * se1 * se2 + (1 - pi) * (1 - sp1) * (1 - sp2), # ++
      pi * se1 * (1 - se2) + (1 - pi) * (1 - sp1) * sp2, # +-
      pi * (1 - se1) * se2 + (1 - pi) * sp1 * (1 - sp2), # -+
      pi * (1 - se1) * (1 - se2) + (1 - pi) * sp1 * sp2  # --
    )
  }

  p1 <- prob_cat(pi1)
  p2 <- prob_cat(pi2)

  # Add small epsilon to avoid log(0)
  eps <- 1e-12

  -(
    sum(counts1 * log(p1 + eps)) +
    sum(counts2 * log(p2 + eps))
  )
}

# --- Initial parameter guesses ---

init <- c(
  logit(0.5), # logit_pi1
  logit(0.2), # logit_pi2
  logit(0.9), # logit_se1
  logit(0.9), # logit_sp1
  logit(0.9), # logit_se2
  logit(0.9)  # logit_sp2
)

# --- Fit model with MLE ---

fit <- optim(
  par = init,
  fn = negloglik,
```

```
  method = "BFGS",
  hessian = TRUE
)

# --- Extract parameter estimates ---

# Transform back from logits
estimates <- plogis(fit$par)
names(estimates) <- c(
  "pi1", "pi2",
  "Se1", "Sp1",
  "Se2", "Sp2"
)

print("MLE Estimates:")
print(estimates)

# --- Compute 95% CI via Hessian ---

# Get variance-covariance matrix
vcov <- solve(fit$hessian)
se <- sqrt(diag(vcov))

# Delta method: CI on probability scale
ci_lower <- plogis(fit$par - 1.96 * se)
ci_upper <- plogis(fit$par + 1.96 * se)

# Display CIs
results <- data.frame(
  Parameter = names(estimates),
  Estimate = estimates,
  CI_lower = ci_lower,
  CI_upper = ci_upper
)

print("95% CI via Hessian:")
print(results)

# --- Bootstrap for robust CIs ---

set.seed(123)
B <- 1000 # Number of bootstrap iterations
boot_results <- matrix(NA, nrow = B, ncol = length(estimates))

for (b in 1:B) {
  # Bootstrap resample counts via multinomial
  boot_counts1 <- as.vector(rmultinom(1, sum(counts1), prob = counts1 / sum(counts1)))
  boot_counts2 <- as.vector(rmultinom(1, sum(counts2), prob = counts2 / sum(counts2)))

  # Define new likelihood with bootstrapped data
  negloglik_boot <- function(
    logit_pi1, logit_pi2,
    logit_se1, logit_sp1,
    logit_se2, logit_sp2
  ) {
    pi1 <- plogis(logit_pi1)
    pi2 <- plogis(logit_pi2)
    se1 <- plogis(logit_se1)
    sp1 <- plogis(logit_sp1)
    se2 <- plogis(logit_se2)
    sp2 <- plogis(logit_sp2)

    prob_cat <- function(pi) {
      c(
        pi * se1 * se2 + (1 - pi) * (1 - sp1) * (1 - sp2),
        pi * se1 * (1 - se2) + (1 - pi) * (1 - sp1) * sp2,
        pi * (1 - se1) * se2 + (1 - pi) * sp1 * (1 - sp2),
        pi * (1 - se1) * (1 - se2) + (1 - pi) * sp1 * sp2
      )
    }

    p1 <- prob_cat(pi1)
    p2 <- prob_cat(pi2)

    eps <- 1e-12
```

```
    -(
      sum(boot_counts1 * log(p1 + eps)) +
      sum(boot_counts2 * log(p2 + eps))
    )
  }

  # Re-fit to bootstrap sample
  boot_fit <- try(optim(
    par = fit$par,
    fn = negloglik_boot,
    method = "BFGS"
  ), silent = TRUE)

  # Store if successful
  if (inherits(boot_fit, "try-error")) next

  boot_results[b, ] <- plogis(boot_fit$par)
}

# Remove failed iterations
boot_results <- boot_results[complete.cases(boot_results), ]

# Compute bootstrap CIs
boot_ci_lower <- apply(boot_results, 2, quantile, probs = 0.025)
boot_ci_upper <- apply(boot_results, 2, quantile, probs = 0.975)

# Display bootstrap CIs
boot_results_df <- data.frame(
  Parameter = names(estimates),
  Estimate = estimates,
  Boot_CI_lower = boot_ci_lower,
  Boot_CI_upper = boot_ci_upper
)

print("Bootstrap 95% CIs:")
print(boot_results_df)

# --- Helper: Logit and inverse logit ---

logit <- function(p) log(p / (1 - p))
```

🚀 Summary of Features in This Code:

✅ Latent Class Model of Hui & Walter
✅ Two tests, two populations
✅ Logistic reparametrization
✅ MLE via `optim()`
✅ Hessian-based confidence intervals
✅ Parametric bootstrap with multinomial resampling
✅ Modular code to easily adapt to your data

⚠️ Notes

- You should **replace `counts1` and `counts2`** with your actual data.
- The **bootstrap takes time** for `B=1000` — you can reduce `B` while testing.
- The **logistic parametrization** guarantees that Se, Sp, and prevalences stay between 0 and 1.

If you'd like, I can also provide:
✅ **Visualization of the bootstrap distributions**
✅ **Model checking diagnostics**
✅ **Extension to >2 tests or >2 populations**

Would you like me to generate a full **worked example with simulated data** and **plots** to illustrate the method further? Just say **yes** and I'll prepare it! 🚀
